# Crop Adaptation: Weedy and Crop Wild Relatives as an Untapped Resource to Utilize Recent Increases in Atmospheric CO_2_

**DOI:** 10.3390/plants10010088

**Published:** 2021-01-04

**Authors:** Lewis H. Ziska

**Affiliations:** Mailman School of Public Health, Columbia University, New York, NY 10032, USA; lhz2103@cumc.columbia.edu

**Keywords:** adaptation, breeding, CO_2_, CWR, seed yield

## Abstract

Adaptation measures are necessary to ensure the stability and performance of the food supply relative to anthropogenic climate change. Although a wide range of measures have been proposed (e.g., planting dates, crop choices, drought resistance), there may be a ubiquitous means to increase productivity relatively quickly. Numerous studies have shown that the projected increase in atmospheric CO_2_ can stimulate crop growth and seed yield with noted intra-specific differences within crop cultivars, suggesting potential differences to CO_2_ that could be exploited to enhance seed yield in the future. However, it is worth emphasizing that atmospheric CO_2_ has already risen substantially (≈27% since 1970) and that, at present, no active effort by breeders has been made to select for the CO_2_ increase that has already occurred. In contrast, for weedy or crop wild relatives (CWR), there are indications of evolutionary adaptation to these recent increases. While additional steps are needed, the identification and introgression of these CO_2_-sensitive traits into modern crop cultivars may be a simple and direct means to increase crop growth and seed yield.

## 1. Introduction

Maintaining food security is a seminal objective for the remainder of the century. While there are a number of recognized obstacles to achieve this objective, environmental limitations associated with unprecedented anthropogenic change threaten core aspects, including production, access, and quality. Specifically, climate change can alter abiotic environments, such as water availability (too much or too little), irregular temperatures, and extreme climatic events (flash droughts, derechos, etc.) [1,2]. Climatic-induced changes in biotic competition from agronomic pests (insects, disease, weeds) pose another significant constraint to global food production [3,4].

Such vulnerabilities within the agronomic food chain necessitate an immediate need to begin adapting crops to empirical threats associated with climatic change. Adaptation per se represents a wide range of approaches, but one facet of obvious consequence is genetics. In that regard, there are a number of exemplary and ongoing efforts to select for crop lines that can respond to climatic extremes, such as drought or extreme temperature [5,6,7].

There is another genetic approach that is becoming recognized as a potential adaptation tool: the selection of intra-specific variation in seed yield among C_3_ crop cultivars in response to projected increases in atmospheric CO_2_. Such an approach is being used to determine CO_2_ sensitivity in conjunction with economic yield for cassava [8], rice [9], soybean [10], wheat [11], inter alia. At present, there is ample evidence that considerable variation exists within crop cultivars for anticipated increases in CO_2_ and that selection for such variation holds promise as an adaptive means to increase crop yields.

Yet, the anticipated increase in atmospheric CO_2_ is, in the short term, slow, 2–3 ppm per year, and selection for cultivars for projected CO_2_ levels 30–50 years into the future does not address the current need to adapt crop systems.

On the other hand, atmospheric CO_2_ has already increased substantially from ≈325 to 412 ppm since 1970, which is an increase of ≈27%. Has this recent increase been exploited through ongoing artificial selection to choose current crop lines that are CO_2_ sensitive? Has the increase in CO_2_ been sufficient to begin evolutionary selection for increased growth and seed yield for weedy or crop wild relatives (CWR)? The objective of the current review is to compare and contrast selection efforts from breeders and nature with respect to CO_2_ sensitivity and potential seed yield for these two groups and to provide insight into metrics that could be of immediate (and future) benefit for utilizing CO_2_ to increase crop growth and seed yield.

## 2. Breeder Efforts to Select for CO_2_ Responsiveness in Crops

While intra-specific variation in response to future CO_2_ is being evidenced experimentally, there is no verification of any directed attempts by breeders to select for seed yield sensitivity to the increase in CO_2_ that has already occurred. It has been proposed that rapid-cycle breeding and new cultivar introductions could, over time, incrementally improve crop lines in adapting to new climates [12]. If true, then breeders could be already selecting (albeit passively) the most CO_2_-responsive cultivars over time. As such, there would be little need to initiate any active CO_2_ breeding programs to exploit recent (or projected) changes in CO_2_ with respect to seed yield.

Has such an approach worked to date? If modern, recently introduced lines are adapted to current CO_2_ levels (≈412 ppm), then they should demonstrate a greater CO_2_ response to recent CO_2_ increases relative to cultivars that were developed during the early twentieth century.

There are a number of studies that have explicitly tested this question. Sakai et al. [13] examined five japonica rice lines: ‘Aikoku’ (released in 1882), ‘Norin 8ʹ (1934), ‘Koshihikari’ (1956), ‘Akihikari’ (1976), and ‘Akidawara’ (2009). No differences in seed yield were noted between the oldest line (Aikoku, 1882) and the newest line (Akidawara, 2009) to increased CO_2_ (19 and 19.3%, increase at 600 ppm CO_2_, relative to ambient, respectively). For wheat, yield responses to rising CO_2_ actually declined with the release of newer cultivars [14,15]. For oat, Ziska and Blumenthal [16] examined the growth and vegetative characteristics of cultivated oat (*Avena sativa* L.) from seven geographical locations to CO_2_ concentration increases that corresponded roughly to the CO_2_ from the 1920s (300 ppm), as well as current (400 ppm) and mid-21st century projected levels (500 ppm). Newer lines were less responsive than older lines to rising CO_2_ in terms of both leaf area and tiller number. Overall, there is little evidence that cultivars in current use are those best adapted to maximize productivity in response to increasing atmospheric CO_2_ (but see [17] for barley).

Interestingly, for the oat study [16], significant age × CO_2_ interactions were observed with greater phenotypic variation noted for the older cultivars (Table 1). Diminished variance and increasing genetic uniformity is not unexpected, given increased farm size and greater mechanization during the 20th century [18]. However, uniformity can also limit responses to environmental factors, including rising CO_2_ [19], suggesting that the ability to respond to rising CO_2_ or other environmental perturbations may be constrained through modern breeding efforts. Overall, the narrow genetic base of modern cultivars may constitute a major bottleneck for crop improvement efforts [20,21,22].

## 3. Weedy and Wild Crop Relatives

Crop wild relatives (CWR) are those undomesticated “cousins” of cultivated crop lines that represent a potential untapped genetic resource that could be used as a means to adapt to new pests or abiotic changes, including, at least theoretically, increased levels of CO_2_. Of course, there are no directed efforts to adapt CWR to the recent increase in CO_2_; however, it is worth determining if any evolutionary adaptive changes that have allowed CWRs to adapt to recent CO_2_ increases have occurred.

It can be argued that such evolutionary changes are unlikely given the recent increase in CO_2_. However, the traditional paradigm of weed evolution as a very slow process is incomplete, and there are a number of examples demonstrating that rapid evolutionary change (years or decades) can occur within weed biology, (e.g., *Microstegium vimineum*, [23]; *Lythrum salicaria*, [24]; *Brassica rapa*, [25]; *Avena fatua*, [26]). In turn, such changes could include evolution in response to anthropogenic climate change [27,28,29].

At present, there is initial evidence indicating that recent increases in CO_2_ may have already altered the adaptive response of some annual weeds. For example, Bunce [30] examined recent increases in CO_2_ on the growth response of four annual weeds over a narrow CO_2_ range (90 ppm below and above ambient) and demonstrated that the efficiency by which these weeds utilized CO_2_ declined at concentrations above ambient, indicating that these weeds had adapted to recent CO_2_ increases.

**Figure 1:**
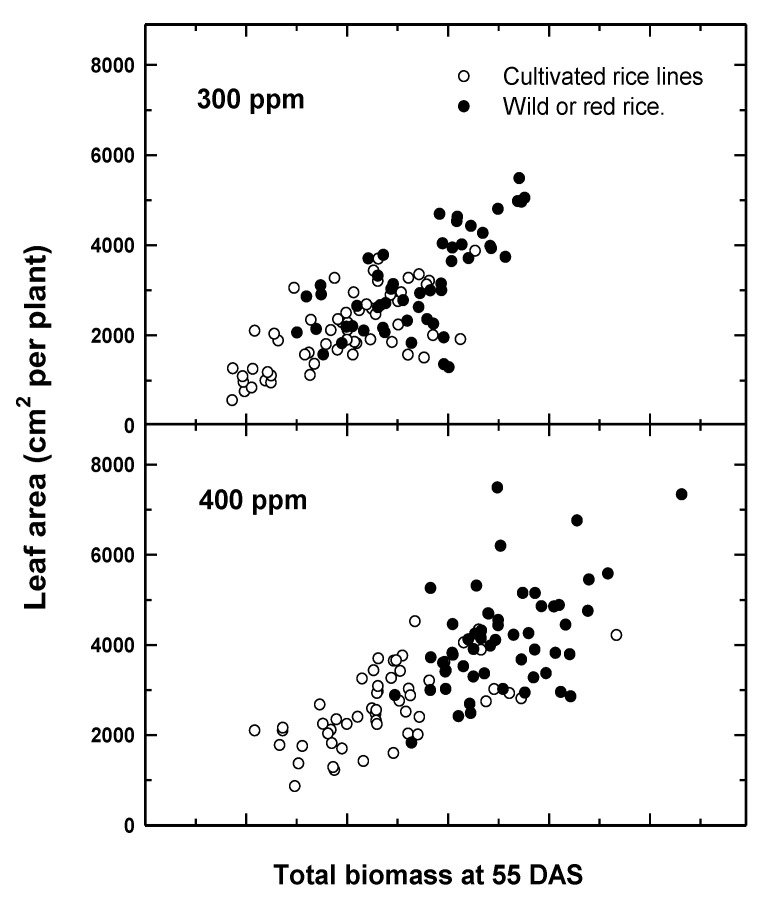
Response of six red rice (RR, filled circles) and six cultivated rice (CR, open circles) varieties to the recent change in atmospheric CO_2_ concentrations (300–400 ppm) for leaf area as a function of total above-ground biomass 55 days after sowing. Note the increase in growth for RR for the recent CO_2_ increase. Data are adapted from Ziska and McClung [31].

With respect to CWR, comparisons of relative fitness to cultivated lines suggest differential adaptation to recent CO_2_ increases. Comparisons of six cultivated and six wild or weedy biotypes of rice indicated a greater overall growth response among wild relative to cultivated rice to recent (300–400 ppm) increases in CO_2_ [31] (Figure 1), suggesting that the rapid evolution of weedy biotypes may have increased their fitness relative to the crop. Greater seed yields were also recorded for Stuttgart, a CWR to rice, compared to Clearfield, a cultivated rice line, for the same recent CO_2_ increase [32]. Similarly, using a resurrection approach [33], seeds of two temporally distinct populations of CWR wild oat (*Avena fatua* L.) from the same location, one from the 1960s and one from 2014, (a relative CO_2_ increase of 80 ppm, or 25% from 1960) demonstrated different competitive abilities against a cultivated oat (*A. sativa*) line, with the more recent (2014) *A. fatua* population having greater growth and competitive ability at current CO_2_ levels [34].

## 4. Differences in Selection

Additional data to confirm and expand upon these results is obviously needed. However, it is important to recognize differences within the selection process for cultivated and CWR biotypes. Weeds and wild crop relatives have been subject to natural environmental perturbations for millennia and have as a consequence maintained a much higher level of genetic diversity [21]. Both are characterized by rapid growth, high seed production, environmental plasticity, and genetic variability, and they are considered highly adaptable [35,36]. Such plant species, with short generation times and high seed production, generally show more rapid rates of molecular evolution [37].

Conversely, it is generally recognized that advances in plant breeding are associated with recurrent selection, usually in field environments. As a consequence, selection for say, pest resistance, should be occurring concurrently with rising CO_2_, and, as a result, reflect CO_2_ adaptation. However, plant breeding is a long-term process that can extend over decades, and indirect selection for yield under field conditions is likely to be inefficient because yield is related to a number of abiotic and biotic factors. The unintended consequences of recurring selection are that genetic variation and associated phenotypes can be reduced relative to the available hereditary potential.

Genetic exchange among breeders has also shifted over time. As documented by Atlin [12], prior to the 1990s, breeders typically exchanged varieties. However, as Genetically modified organism (GMO) lines were introduced and breeding became more commercialized, Intellectual Property (IP) protection increased, resulting in the current U.S. practice of issuing utility patents that prevent proprietary lines from being used as parents by other breeders. Unfortunately, simultaneous with these changes, a number of countries recognized that their own indigenous crops were unique genetic resources and restricted their usage in public breeding programs. Consequently, obtaining elite or novel varieties internationally has become restrictive. Overall, such actions have reduced reliance on publically available seed sources, reducing genetic diversity [38].

Given the need for the mechanization of large land holdings and economic consistency in response to water and fertilizer so as to achieve high crop productivity, dedomestication and genetic uniformity are necessary, even if such practices lead to “Domestication Bottlenecks” [39]. However, such uniformity in management may also limit the extent of genetic variation in response to environmental changes, such as CO_2_ [40].

Overall, opportunities for increasing production may be missed if we assume that current breeding efforts have resulted in crop plants that are adapted to the recent increase in CO_2_ concentration. Rather, it suggests that CWR and natural selection may serve as a starting point for active intervention to enhance genetic diversity to meet new environmental uncertainty and optimize crop yields to ongoing CO_2_ increases. In addition, land races, populations of a cultivated plant that are distinct to a given geographic and environmental locale, are worth additional evaluation and may also provide a useful genetic resource in that regard.

## 5. Challenges and Next Steps

If CWR represent an opportunity to exploit the recent increase in CO_2_, it is an opportunity that includes a number of pragmatic challenges. What follows is by no means inclusive but rather representative of next steps.

### 5.1. Phenotypic and Genotypic CO_2_ Sensitivity Traits

Greater effort is necessary to document and determine those traits that may have already contributed to a greater sensitivity to recent CO_2_ increases (e.g., red rice). A consensus is needed to identify those phenological, morphological, and/or physiological characteristics that are associated with CO_2_ responsiveness. Initial studies have suggested different organismal levels associated with CO_2_ sensitivity including genetic (e.g., carbohydrate regulation of RNA, [41], biochemical (e.g., Rubisco activase) [42], leaf (e.g., stomatal density [43] or photosynthesis [44], whole plant (relative growth rate) [45], management (e.g., planting density) [46] and canopy (e.g., nitrogen applications) [47], but specific organismal characteristics consistently associated with CO_2_ responsiveness and crop yield for CWRs have not been identified. A definitive set of these parameters is necessary for breeders to select CO_2_-sensitive crop archetypes.

### 5.2. Introgression of CWR Traits

While CWRs can make an effective contribution to broadening the genetic diversity of crops, their direct use in breeding has primarily focused on introgressing loci for disease resistance, not abiotic stress [48,49]. This is due, in part, to the presence of objectionable traits in CWRs (e.g., transfer of undesirable QTLs) as well as breeding barriers with the crop.

Initiatives have commenced that aim to adapt agricultural sustainability to climate change through the use of CWRs to broaden and improve the cultivated gene pool as part of the International Treaty on Plant Genetic Resources for Food and Agriculture (ITPGRFA, [50]). This effort is designed to collect, preserve, and prepare CWRs for evaluation and potential adaptation of crops to climate change. Other initiatives, such as Diversity Seek (DivSeek) are underway to begin evaluating the potential of crop and wild relative diversity present within gene banks [51].

To overcome difficulties in facilitating gene introgression, Prohens et al. [52] have suggested a novel approach, ‘introgressiomics’, a mass-scale expansion of plant materials and populations that confer genetic introgressions from CWRs into crops. Their goal is to generate chromosome substitution lines (CSLs), introgression lines (ILs), and multi-parent advanced inter-cross (MAGIC) populations through the use of marker-assisted selection as a means to characterize genetic traits present in CWRs, but to also develop genetically relevant elite materials that can be incorporated into breeding programs as a means to adapt crops to climatic change.

### 5.3. Additional Climatic Variables

The climatic consequences of increasing CO_2_ are obvious and include increases in surface temperature, changes in precipitation and extreme events that will have negative consequence for crop productivity. Temperatures, especially during anthesis, may be critical in maintaining yield performance [53]. In addition, there are numerous studies indicating that rising temperature per se may negate any stimulatory effect for projected CO_2_ increases [54,55,56].

At present, evaluations of CWR to both recent increases in CO_2_ and temperature are unavailable; however, it is of interest to note one seminal study that has examined Indica, Indica-like, Japonica, and CWR of rice to projected CO_2_ concentrations four temperature treatments [57]. They reported an increased yield sensitivity to high temperature stress at higher (600 ppm) CO_2_, but their results also showed that CWR for rice demonstrated superior CO_2_ x temperature interactions with respect to yield, supporting the idea of using wild or unadapted gene pools in rice to enhance breeding efforts for climate change adaptation.

In any case, a fundamental challenge in CO_2_ selection will be to consider multiple environmental interactions with a merited focus on temperature and moisture conditions to assess possible negative interactions with respect to yield. By necessity, such selections will include evaluations of multiple-gene responses.

### 5.4. Nutritional Considerations

For projected CO_2_ increases, there is considerable evidence indicating that stimulatory effects of increased CO_2_ may be accompanied by declines in nutritional quality, including but not limited to protein and minerals [58,59,60,61]. The basis for the CO_2_-induced changes in crop quality are still being elucidated, in part because there are a number of biological and physical processes that are influenced by increasing CO_2_ [62]. However, any efforts to adapt CWRs and cultivars to recent CO_2_ increases must include concurrent selection efforts or the co-development of suitable management practices that will maintain the desired quality and nutritional characteristics necessary for human health.

### 5.5. Scaling Up to the Field Level 

Genomic and molecular traits that are assimilated into new cultivars may increase performance at the whole plant or leaf level; however, as emphasized by Sinclair et al. [63], such improvements need to scale up to field responses. As such, the identification of CO_2_-induced increases in the photosynthetic rate or seed yield of single leaves or whole plants in the laboratory require complementary management approaches that will illicit similar responses in the field. In this regard, it may be worthwhile to examine historical trends by cultivar. For example, comparisons of yield performance from 1950s cultivars, relative to the same cultivars under today’s CO_2_ concentration using similar metrics (row spacing, soil types, pesticide usage) could help identify cultivar x CO_2_ sensitivity and, potentially, elucidate management practices that could maximize CO_2_ yield responses in situ.

## 6. Conclusions

The challenge of adapting to an uncertain climate is paramount to maintaining global food security. High yielding crop varieties with tolerance to biotic and abiotic stresses associated with climatic change are needed to meet this challenge. Currently, there is recognition that the current genetic base of modern cultivars may be too narrow for crop improvement efforts in that regard. In turn, this may reflect a lack of genetic ideotypes that encompass traits associated with climatic change or uncertainty associated with the rate of change. As such, the use of CWR (or landraces) may be a means of enhancing genetic diversity of cultivated crops as a means to adapt to these changes.

Among adaptation efforts, there is an opportunity to exploit the recent increase in atmospheric CO_2_ as a means to stimulate plant growth and yield. Currently, comparisons of cultivated and CWR indicate differential selection in regard to this change, with initial evidence suggesting evolutionary adaptation for some CWR, e.g., red rice. Introgression of these traits into current cultivars, and appropriate management practices, may provide a means to utilize the increase in CO_2_ that has already occurred (≈27% increase since 1970) to stimulate seed yield.

At present, there is no empirical evidence that breeders are selecting for CO_2_ responsiveness. This may be due, in part, to current economic and agronomic practices associated with modern agriculture. Yet, it seems surprising that CO_2_ as a potential adaptation strategy to maintain global food production is not being utilized. One can ask if other abiotic resources such as sunlight, water, or nutrients had increased to a similar extent in recent decades whether incentives to optimize that increase would be underway.

Adaptation to recent CO_2_ increases will not be a complete solution to the complications associated with climate change. However, adaptation may represent one of the simplest research strategies to help maintain global food security relative to the anthropogenic stresses associated with climatic change. As such, it is hoped that this review can serve as a sounding board and starting point for additional efforts.

## Figures and Tables

**Table 1:** Significance of equality of variance for “old” and “new” oat cultivars for select vegetative and growth characteristics averaged over all CO_2_ concentrations. An asterisk indicates a greater degree of phenotypic variation. Note the greater degree of phenotypic variation for oat cultivars released in the 1920s. Data are from 16.

Variable	“Old”	“New”
Leaf area	*	
Leaf wt.	**	
Tiller wt.	**	
Weight tiller^−1^	***	
Tiller No.	*	
Root wt.	*	
Total wt.	***	
RGR	**	

* *p* < 0.05; ** *p* < 0.01; *** *p* < 0.001. n.s. = not significant.

## Data Availability

The article is a review synopsis and does not contain original data.
